# Quality of Life Assessment Among Ethnically Diverse Black Prostate Cancer Survivors: A Constructivist Grounded Theory Approach

**DOI:** 10.21203/rs.3.rs-3941497/v1

**Published:** 2024-02-20

**Authors:** Motolani Ogunsanya, Ernie Kaninjing, Tanara Ellis, Olufikayo Bamidele, Daniel Morton, Andrew McIntosh, Sabrina Dickey, Darla Kendzor, Kathleen Dwyer, Mary Ellen Young, Folakemi Odedina

**Affiliations:** University of Oklahoma Health Sciences Center; Georgia College & State University; University of Oklahoma Health Sciences Center; Hull York Medical School; University of Oklahoma Health Sciences Center; University of Oklahoma Health Sciences Center; Florida State University; University of Oklahoma Health Sciences Center; University of Oklahoma Health Sciences Center; Mayo Clinic; Mayo Clinic

## Abstract

**Purpose:**

Prostate cancer (CaP) is the most common cancer in Black men (BM), and the number of Black CaP survivors is rapidly increasing. Although Black immigrants are among the fastest-growing and most heterogeneous ethnic groups in the US, limited data exist regarding their CaP experiences. Therefore, this study aimed to explore and model the experiences of ethnically diverse Black men with CaP.

**Methods:**

In-depth interviews were conducted with 34 participants: Native-born BM (NBBM) (n=17), African-born BM (ABBM) (n=11), and Caribbean-born BM (CBBM) (n=6) CaP survivors recruited through QR-code embedded flyers posted in Black businesses, clinics, social media platforms, and existing research networks within the US. Guided by Charmaz’s constructivist grounded theory methodology, the interviews were analyzed using constant comparison following key stages of initial, focused, and theoretical coding using Atlas.ti v23.

**Results:**

Participants were thirty-four men aged 49–84 years (mean±SD, 66±8). Most were married (77%), likely to be diagnosed at Stage I (35%), and treated with radiotherapy (56%). Our study findings explored the complex trajectory of Black prostate cancer (CaP) survivors, unveiling a comprehensive model termed “**Journeying through Unfamiliar Terrain**.” Comprising three phases and 11 sub-phases, this model uniquely captures the pre-diagnosis awareness and post-treatment adaptation among survivors.

**Conclusion:**

The resulting theoretical model delineates the entire CaP survivorship process among BM, providing contextual and conceptual understanding for developing interventions and enhancing patient-centered care for ethnically diverse CaP survivors, pivotal in bridging the gaps in survivorship research and healthcare practices.

## INTRODUCTION

Prostate cancer (CaP) is the most prevalent cancer among men, with Black men (BM) twice as likely as non-Hispanic White men (NHWM) to be diagnosed [1]. Additionally, CaP in BM often presents at more advanced stages, resulting in higher mortality rates compared to other racial or ethnic groups [2]. These disparities have been attributed to multifaceted factors, including genetic susceptibility, reduced access to healthcare, family history, socioeconomic factors, and late detection [3–6]. Despite significant strides in early detection and treatment, the landscape for CaP is rapidly evolving, leading to a decline in mortality rates from 82.1 to 36.6 per 100,000 between 1993 and 2019 [1, 7, 8]. Since the overall 5-year survival for BM with CaP is approximately 97%, a growing population of Black survivors must cope with the physical, emotional, and mental symptoms that persist long after recovery from acute CaP. Transitioning from treatment to survivorship can be burdensome and significantly impact quality of life (QoL) [9–11].

BM are disproportionately impacted by aggressive forms of CaP, leading to significant disparities in post-treatment experiences and long-term outcomes. Post-treatment complications, including functional impairments (e.g., sexual dysfunction, urinary incontinence, and fatigue), negatively impact the quality of life (QoL) [12–16]. Notably, studies have underscored lower levels of physical and emotional functioning among BM compared to NHWM, highlighting disparities in recovery and satisfaction post-treatment [17, 18].

Addressing these disparities necessitates a comprehensive examination of diverse Black populations, including those of African and Caribbean descent, as well as genetically predisposed individuals [19, 20]. Acknowledging the multifaceted factors contributing to these disparities can aid in more equitable survivorship outcomes and enhance the QoL of ethnically-diverse BM men affected by CaP – a relatively understudied area of research. Understanding such impact can guide future interventions aimed at improving the continuum of CaP care from diagnosis to survivorship. For such interventions to be successful, they must be theoretically driven, evidence-based, and culturally sensitive [21]. Therefore, this study aimed to understand the experiences of ethnically diverse Black CaP survivors in the United States using a Constructivist Grounded Theory approach, with the aim of developing a substantive theory in this population. This approach acknowledges survivors’ subjective realities and diverse perspectives, allowing for an in-depth exploration of the multifaceted factors shaping their survivorship experiences, which could inform clinical practice and intervention development [22, 23]. Specifically, we focused on native-born Black men (NBBM), African-born Black men (ABBM), and Caribbean-born Black men (CBBM), aiming to explore the nuances and complexities of their survivorship journeys.

## METHODS

### Study design

This study employed a constructivist grounded theory approach as the methodology, facilitating the co-construction of knowledge through dynamic researcher-participant interactions [22]. This approach was chosen to align with the study’s aim of exploring diverse perspectives and factors influencing survivorship experiences of ethnically diverse men with CaP, ultimately developing an explanatory framework to understand the phenomena under investigation [24, 25].

### Data collection

Between January 2021 and August 2023, adult Black CaP survivors residing in the United States were recruited through QR-code-embedded flyers distributed across Black businesses, clinics, social media platforms, and existing research networks. Inclusion criteria required: 1) Black race, 2) confirmed CaP diagnosis, and 3) ability to read and understand English. Men of other races or those without a history of CaP diagnosis were excluded. Initially, convenience and snowball sampling methods were employed to identify the initial cohort of participants enrolled in the study [22]. Subsequently, to increase the rigor of data collection, theoretical sampling was employed. This method involves selecting samples based on concepts that demonstrate theoretical relevance to the developing theory [26]. Specifically, theoretical sampling was used to explore emerging preliminary findings from the initial data analysis, and subsequent interviews were conducted until saturation was achieved [27]. The findings were reported following the Consolidated Criteria for Reporting Qualitative Research (COREQ) guidelines [28].

The audio-recorded interviews were conducted remotely via Zoom and phone and lasted 48–73 minutes (Mean ± SD, 66 ± 24 minutes). The interviews were conducted by MEO, an African-born female in her mid-30s, and EK, an African-born male in his late 40s, without any pre-existing relationship with the participants. A semi-structured interview guide was used, which was developed based on input from the literature review, Black CaP survivors serving as community advisory board (CAB) members (n = 6), a urology oncologist, and the research team with diverse expertise in community-engaged and CaP disparity research (see Table 1). After the initial interviews, the topic guide was iteratively revised based on emerging themes, ensuring a more detailed exploration of participants’ evolving perspectives and diverse experiences. Demographic and clinical data, including age, ethnic group, stage and length of diagnosis, type of treatment, family history, and marital status, were collected.

### Data analysis

Demographic responses were analyzed using descriptive statistics (e.g., frequencies, means) on SPSS version 23. All interviews were transcribed verbatim by a professional transcription service. The transcripts were analyzed using grounded theory procedures [22, 29, 30]. Initially, data organization facilitated subsequent coding phases. Coding was done by three researchers (MEO, OB, EK) according to the steps outlined by Charmaz [22]. First, “initial coding” helped identify critical concepts within the texts, creating corresponding labels to represent their meanings, while “focused coding” was implemented to compare and group the codes into broader categories. As each interview was coded, existing codes were supplemented with additional data, while new codes and categories were introduced to incorporate emerging concepts.

Throughout the analysis, constant comparison methods [29] were employed, allowing for the establishment of boundaries and contexts from the codes and categories, enabling data comparison within and between interviews. In line with theoretical sampling, simultaneous data collection and analysis enabled comparing codes and data from later interviews, to explore similarities and variations in the men’s experiences until theoretical saturation was achieved (when the research phenomenon was well understood and conceptualized). with those from earlier interviews, affirming the relevance and application of data interpretation. This is linked to the theoretical coding phase, wherein categories were organized to develop the core category which embodied the central theme within the data to understand the study phenomenon [31]. Recorded memos were crafted during data collection, reflecting grounded theory principles and aiding in forming the emerging theory [22]. Multiple meetings were held with the research team and CAB members to deliberate on interpretations and insights derived from the data, culminating in an iterative consensus on identified themes over several weeks. The transcribed data were coded and sorted using the computerized-assisted text analysis software Atlas.ti v23.

The University of Oklahoma Institutional Review Board approved the study (IRB #11441), and informed consent was obtained from all participants.

## RESULTS

After 34 interviews with 17 NBBM, 11 ABBM, and 6 CBBM, theoretical saturation was reached. Participants’ clinical and demographic characteristics are summarized in Table 2. Half of the participants were NBBM, and most were married (76.5%), and had undergone radiotherapy (55.9%).

### Findings

**Figure 1:**
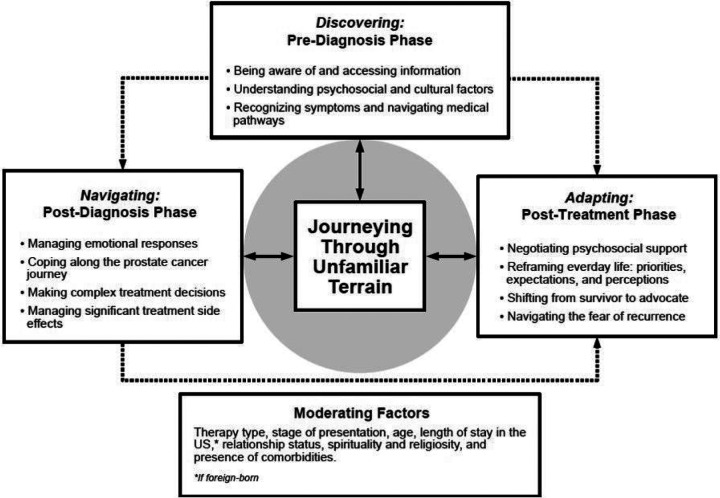
A conceptual model of the experiences of ethnically diverse Black men with prostate cancer

From the depth and richness of conversations drawn from the participants, it became evident that participants had experienced a unique and compelling process of adjustment to their diagnosis, labeled as “**Journeying through Unfamiliar Terrain**” (see Fig. 1). These stages encapsulated the process of *discovering (pre-diagnosis phase), navigating (post-diagnosis phase), and adapting (post-treatment phase)* evident in the diverse personal and shared encounters and insights of ethnically diverse Black CaP survivors.

### Discovering: Pre-Diagnosis Phase

In the Discovering Phase, individuals encountered pivotal challenges and considerations in their journey toward a CaP diagnosis. This phase also involved the journey towards CaP awareness, the level of information available in the community related to CaP, comprehension of the psychosocial and cultural influences, and the events, including symptoms that prompted these individuals to engage with the healthcare system. This phase also profoundly shaped their survivorship journey, establishing the foundation for informed decision-making and empowering steps toward addressing their health concerns.

Participants struggled with lack of information and awareness related to CaP. One individual highlighted this, stating:

“You know, the first thing that came to my mind is outreach. You know, if it was advertised more or if they had…like, in my case, I just happened to come upon a satellite office that offered screenings. And if they had more of these in the Black neighborhood where people could walk around the corner and take the test, it would be beneficial. Up until then, I had not heard about it, but I did not know of, you know, some of the difficulties of dealing with prostate cancer. And chances are, if it had not been there, I wouldn’t have taken the test” – (75 years, NBBM).

This sentiment was further supported by the cultural inclination against sharing family medical histories, as expressed by another participant:

“As a Nigerian, discussing medical histories is not a common practice. In hindsight, I suspect my father had a similar health issue as he struggled with urination before his passing. However, the cause of his death remains a mystery to me – (71 years, ABBM).”

*Understanding Psychosocial and Cultural Factors* delved into the complexities of cultural attitudes and sociocultural taboos surrounding CaP within the Black community regardless of ethnic groups. These complexities often contribute to delayed cancer detection. One participant vividly described this issue:

“Prostate cancer often carries stigma, especially around issues like impotence. Many in the Black community see it as a death sentence. Even though it was caught late in my case, I beat it by being proactive. Once I knew what to do, I was all in, never missing a doctor’s appointment. In fact, I requested more visits – (67 years, ABBM).”

The fear of invasive procedures and complications like urinary issues and erectile dysfunction delayed CaP diagnosis. The stigma around these procedures, influenced by societal perceptions and stereotypes, particularly regarding the digital rectal examination, hindered early diagnosis. This fear, including its association with homosexuality, persisted across ethnic groups, albeit influenced by diverse factors.

It’s more than just religious. It’s a scar from slavery. Jamaican men know they will die; they aren’t worried about that. Jamaicans don’t really like the prostate examination because of the impact of slavery, especially because the slave masters used to sodomize, you know, Jamaican men. So that hatred of homosexuality and anal sex, anything that has to do with anal manipulation, is deeply entrenched in the culture because of that impact on slavery. I just heard yesterday about one of my sister’s sons who has prostate cancer. They know the danger of it, they’ll die, but they’d rather protect their sexual health because being impotent is also like a curse – (63 years, CBBM).

A lot of Black men have a fear of going, getting the prostate checked. I, myself, have feared getting it checked – (75 years, ABBM).

*Recognizing Symptoms and Navigating Medical Pathways* emphasized the significance of identifying pre-diagnostic symptoms such as pelvic and bone pain, sleep disruption from frequent urination, and erectile dysfunction and effectively managing them. One participant recounted,

“What initiated everything was missing meetings due to sleeplessness caused by frequent trips to the bathroom. Explaining this to the director led to involving the oncology team, which led to my diagnosis – (59, NBBM).”

Interestingly, some men experienced no apparent symptoms before diagnosis; one participant stated: *“I didn’t have any symptoms, so I kept up with my annual check-ups. Many avoid the rectal exam, but I didn’t have any symptoms at all” – (63, CBBM)*.

### Navigating: Post-Diagnosis Phase

During this phase, spanning from diagnosis to treatment initiation for CaP, individuals experienced a myriad of complex emotions ranging from bargaining and acceptance of diagnosis, to managing treatment decisions. Decision-making became pivotal, often influenced by psychosocial support, marital status, and the patient-provider relationship. Coping mechanisms also varied, shaped by stigma and the availability of reliable information, often through the internet and healthcare providers. Thus, relationships with healthcare providers become crucial in navigating these complexities, while caregivers play indispensable supportive roles. The navigation through these aspects characterizes the coping processes in the CaP journey. Managing significant treatment side effects that impact quality of life also becomes critical in this phase.

A positive diagnosis of CaP was followed by a broad spectrum of emotions, with participants reporting that they experienced fear, anxiety, and uncertainty. Consequently, many said that they leaned into their community and other existing networks for support. For example,

I am active on Facebook and WhatsApp. I talk with my friends, you know. These are some of the things that keep me, you know, keep me sane – (66 years, ABBM).

Some participants reported utilizing other coping strategies, which included active research via the internet to gather information about CaP, connecting to a higher power, making dietary adjustments, and increasing physical activity levels to promote longevity, and exploring complementary and alternative medicine. For example,

“My faith is my cornerstone, my unwavering support. I firmly believe in God, knowing He’s in control of everything. I have no fear of dying; I trust He will heal me of my cancer – (66 years, ABBM).”

It is worth noting that foreign-born participants, particularly those from Africa, faced additional challenges due to acculturation and dietary changes influenced by cultural factors, complicating the implementation of recommended dietary modifications for CaP management.

Mostly, I like eating fruits, like oranges, bananas, and some stuff like rice. You know, I like eating our African food like fufu, but my primary doctor was saying that a lot of that needed to change. It was very difficult – (59, ABBM).

Peers, family, and healthcare providers were vital in guiding treatment decisions. Participants revealed they were aided through treatment options and received invaluable support from the experiences shared by other survivors. Trusted healthcare providers were also instrumental for their expertise and reassurance, where an established relationship with primary care providers was linked with better satisfaction in treatment outcomes. Consistent healthcare and relationships with primary doctors led to earlier cancer diagnoses, specialist referrals, and effective post-treatment care. Notably, foreign-born Black men tended to establish long-lasting relationships with healthcare providers sharing their racial and ethnic backgrounds.

My primary doctor, who was with me when it was discovered, has retired, but I have another great African one, and I love our relationship. We always have friendly conversations, and we talk to each other. And I feel that I’m talking to someone who cares about me and could keep me as healthy as possible, so I trust him, and I have annual checkups with him – (84, ABBM).

Interestingly, the study also suggested ethnic differences in decision-making dynamics. Native-born BM tended to involve their spouses actively. In contrast, foreign-born BM, especially those of African origin, often informed their spouses after making decisions rather than actively engaging them. For instance, a participant shared,

I didn’t tell my wife and kids about my therapy because I didn’t want to worry them. When my wife arrived, she only found out then. I had already finished the therapy when she found out, and I reassured her that I was okay. She asked me why I hadn’t told her, and I said I was okay, and she could see that for herself – (66, ABBM).

Treatment choices were also made with consideration for factors like stage and grade of cancer, age, and possibility of recurrence. The rationale behind these decisions was to maximize the chances of success in the event of cancer recurrence.

The doctors were telling me, look, if you take it out (i.e., remove the prostate), if it comes back, it’s going straight to your bone. So, please don’t take it out. That’s why I had radiotherapy – (70, ABBM)”.

Men who underwent treatment grappled with diverse treatment side effects, both acute and chronic. Acute effects prevalent during active treatment included fatigue, nausea, pain, and urinary changes. Even post-treatment, chronic issues persisted, like depression, sexual dysfunction, and hormonal imbalances. For many, sexual dysfunction posed the most significant challenge, impacting both themselves and their partners.

If it wasn’t for the penile implant I have, I honestly don’t know what my mental state would be right now. I was very depressed. Would I have committed suicide? Probably not. And I think that’s one of the challenges faced by Black men. They worry, especially about their sexual function. Every Black man I’ve encountered, even in the groups and meetings we used to have at the college, had concerns about their penis. Every single one of them, regardless of their race – (52, NBBM)

Adjusting expectations became vital post-treatment, with some men attributing these changes to aging rather than solely to CaP, aiding their adaptation and overall well-being:

It affects my life because, like my sexual life and that kind of thing, I just take it to be a normal process of aging – (72, CBBM).

Notably, younger men or those with younger partners/wives seemed to be more affected by these sexual changes, intensifying the challenges they faced in managing their intimate relationships.

“Not being able to perform sexually has impacted my life immensely. It’s been years, and at times, it gets frustrating. My wife is still quite young, just in her late 50s. That’s the part that doesn’t sit well with me – (63, ABBM).”

Men undergoing active surveillance experienced considerable anxiety and fear, likening their situation to living with a ticking time bomb. One participant vividly expressed this sentiment, stating:

It’s like living in constant uncertainty, waiting for something to happen, not knowing when or if the bomb will go off – (65, ABBM).

“So, that’s the other thing I always worry about because I went celibate many years ago. I went celibate when we started talking about prostate cancer. Part of it is because I thought I could infect my wife with cancer. I was ignorant of it. I didn’t want anything about me to get into her and cause her issues. Our relationship is a lot more than sexual intercourse, and we will survive without it. Then, it’s not a subject that we talk about. I worry sometimes that because I don’t talk about it anymore…(65, ABBM)”

### Adapting: Post-Treatment Phase

This phase signifies the transition following the post-diagnosis period in the CaP journey. Within this phase, individuals negotiate psychosocial support networks and reframe their everyday lives, altering priorities, expectations, and perceptions. Moreover, they navigate the shift from survivor to advocate, actively engaging in raising awareness or support for others affected by prostate cancer. Additionally, for those who have undergone treatment, there’s the challenge of managing the fear of recurrence, a concern that lingers despite completing initial treatments.

Negotiating psychosocial support took different forms, directly and indirectly. Some found strength in spirituality or faith as a cornerstone of resilience. On the other hand, the support also came from more communal sources like religious congregations or community gatherings, even if the individual chose not to explicitly discuss their cancer journey within such settings, as one man highlighted:

I didn’t directly seek support from anyone. I found solace in the scriptures during that time. I don’t think this is something I should talk about in church. Even though you understand that, you know, a church is a place of refuge – (65, NBBM).

Peer and family support also aided participants post-diagnosis/post-treatment, fostering an environment where survivors could share their experiences. Peer and family support emerges as another critical element in this phase. One participant expressed the significance of this network, saying:

“I think I’ve got a pretty good support system, especially with my family and close friends − (75, NBBM).”

The men in our study faced unique challenges and experiences, and they were also confronted with the need to reassess their priorities in various aspects of their lives, including relationships, work, and personal goals. Despite the profound impact of CaP diagnosis and treatment on their lives, most men in our study expressed deep gratitude for surviving the experience. Many recounted stories of losing friends to CaP, emphasizing the significance of their survival. Being in the United States was viewed as a blessing among foreign-born men, particularly those from low-resource countries. Access to healthcare and advanced medical technologies were seen as instrumental in their successful outcomes, highlighting the valuable role of healthcare resources in improving their chances of survival.

Assume I had been diagnosed in Nigeria; it could have been a different story. I know most of the doctors know that they cannot do it right there, but in Nigeria, we don’t have the equipment to do, you know, to do what needs to be done. So, being here to me is a blessing that helped me out. I feel lucky to be alive because I have lost friends back home to this disease – (60, ABBM).

As men aged and transitioned past the active phase of treatment and post-diagnosis, a common sentiment emerged among them: the desire to become strong advocates for other men in the community; having personally experienced the challenges and uncertainties of CaP, they felt a deep sense of responsibility to share their knowledge, offer support, raise awareness about the disease, and the importance of knowing one’s history. Engaging in advocacy activities provided men with a renewed sense of purpose and prompted them to take more proactive measures for their health:

Yeah, I started a support group here, throughout the state of Florida. I had it going for about seven years – (66, NBBM).

I try to tell them by word of mouth at the barber shop. We’ve got information pamphlets and everything they can pick up. I always try to talk about it – (63, NBBM).

Among those who had undergone therapy, navigating the fear of recurrence emerged as a profound struggle for many. While some were vocal about their concerns, seeking comfort and advice, others carried this worry silently. This fear also propelled survivors towards being proactive about their health via regular physical check-ups and routine monitoring of their prostate-specific antigen (PSA) levels, recognizing the importance of ongoing vigilance and self-care health monitoring.

Many who had undergone therapy found navigating the fear of recurrence to be a significant challenge. This concern also played an essential part in the initial treatment decision-making process. While some openly voiced their worries, seeking comfort, others carried this worry silently. “*I try not to dwell on it, but it’s always there, at the back of my mind,*” confessed a participant, capturing the essence of this pervasive unease. Additionally, this fear motivated survivors to take proactive steps, such as regular physical checkups and monitoring PSA levels. They recognized the importance of ongoing vigilance and self-care for their health.

I gotta see him. That’s a yearly thing with him now. And I have been monitoring my PSA (blood test for CaP) level every six months – (52, NBBM).

Figure 1. A conceptual model of the experiences of ethnically diverse Black men with prostate cancer

## DISCUSSION

This study explored the experiences of ethnically diverse Black CaP survivors, creating a model that characterizes the positive and negative processes shaping their overall survivorship, thus filling a gap in the existing literature. The resulting theory, “**Journeying through Unfamiliar Terrain**,” includes three phases and 11 sub-phases that capture the journey through CaP survivorship. Each stage within the model outlines distinct yet interconnected sub-phases, illustrating the evolution and complexities of experiences in navigating the challenges of survivorship.

The first phase, the *Discovering Phase*, represents a starting point for survivors which begins when men learn about their diagnosis —a distinctive theoretical addition that distinguishes this study from existing studies [32, 33]. In addition, this phase describes the factors that influence awareness, healthcare-seeking behaviors, and cultural perceptions surrounding CaP within Black communities. The model comprehensively describes the moments preceding the confirmation of cancer but also the myriad factors that shape awareness, healthcare-seeking behaviors, and cultural perceptions concerning CaP within Black communities. Within this phase, individuals encountered multifaceted challenges, complexities, and pivotal considerations that set the tone for their entire survivorship journey. This phase also highlighted the importance of awareness and accessibility of information vital for early detection. Further, encouraging dialogue about family medical histories is crucial, considering cultural variations in sharing such information, for example, as reported in some foreign-born participants’ perspectives. Perceptions and attitudes towards CaP within the Black community greatly influence healthcare behaviors, with the stigma surrounding the effects of treatment potentially affecting proactive care-seeking actions [34, 35]. Historical trauma, exemplified by references to slavery, further adds complexity to community reluctance towards specific medical examinations [36–38]. Our study findings also underscore the significance of recognizing the onset of symptoms in CaP and its implications for early detection [39]. Interventions, including education and awareness campaigns, could address misconceptions, stereotypes, and cultural factors influencing fear and stigma. Culturally sensitive approaches and open communication with healthcare providers can also be adopted to promote timely CaP diagnosis, especially in asymptomatic men who have a higher likelihood of being diagnosed with CaP [40].

The Navigating Phase, leading up to a CaP diagnosis, entails emotional, social, and psychological challenges [41, 42]. The emotions reported by individuals post-diagnosis, such as fear, anxiety, and a profound sense of uncertainty, are consistent with findings from other studies [41, 42]. Coping strategies, including spirituality, played a significant role, which was unsurprising as over 88% of participants reported a religious affiliation, finding comfort and guidance through faith and prayer. The critical role of peers, family, and healthcare providers in guiding individuals through their treatment choices is widely documented [43, 44]. Peer support, especially from other CaP survivors, offered valuable shared experiences [45, 46]. In addition, the preference for primary healthcare providers sharing a similar ethnic background among foreign-born participants aligns with research emphasizing racial concordance in patient-provider relationships, which may improve health communication and outcomes [47, 48]. Our study findings highlight ethnic differences in decision-making dynamics, emphasizing the cultural nuances in treatment choices. Familial involvement, especially spousal involvement in treatment decisions, is crucial as it can impact genetic susceptibility and risk assessment within the family [44, 49–52]. Various factors also influenced treatment choices, stressing the importance of personalized care plans and aligning with current medical best practices [53]. Some men faced challenges discussing CaP diagnosis and treatment with family members, possibly due to cultural factors, stigma, or personal preferences. Healthcare providers should offer support and resources to promote open discussion dynamics to ensure that the treatment process is collaborative and supportive for all stakeholders.

Acute and chronic side effects from treatments are major concerns for patients. Sexual dysfunction has been identified as a significant issue impacting QoL [14, 54, 55], which mirrors the concerns most voiced by participants in this study, and it often led to feelings of guilt toward their spouses or partners. The impact of CaP treatment on sexual function not only affected the men themselves but also their intimate relationships. Spouses or partners may experience feelings of frustration, sadness, or disappointment due to the changes in their sexual relationship. The psychosocial impacts of such chronic side effects necessitate comprehensive post-treatment care and support systems [56, 57]. Finally, the anxiety highlighted by men on active surveillance adds to an already growing body of literature on the psychological impact of watching and waiting approaches to cancer treatment [58] and the need for in-depth patient education and psychological support in this delicate period.

*The Adapting Phase* represents a gradual process where individuals move beyond the immediate effects of CaP diagnosis and treatment. It also involved how participants reshaped their day-to-day lives and coping with the long-term implications of their journey with CaP. Our study also confirms the importance of diverse sources of psychosocial support, a factor extensively acknowledged in the literature for its significance in cancer survivorship [54, 57–60]. For example, spirituality and faith were frequently cited by participants as a critical source of support. This support, acknowledged both directly and indirectly, wasn’t always openly deliberated in communal discussions but rather embraced as a personal comfort, highlighting the multidimensional role faith communities play in the lives of cancer survivors [59]. Further, the decision of some participants to seek solace without actively participating in open conversations within faith-based gatherings reflects a nuanced approach to support—the existence of a supportive community might be just as important as direct dialogues about their challenges [60]. The significance of family and peer support parallels findings from the literature [45, 60], which suggests that these social networks provide practical and emotional sustenance, essential in the transition phase post-treatment. In addition, the value placed on peer support reflects convergent dynamics in cancer survivorship experiences, fostering shared experiences that enhance coping strategies [56, 61].

CaP diagnosis fundamentally alters life perspectives, warranting a reevaluation of priorities as described in previous studies [62, 63]. This reevaluation was more poignant among foreign-born men from countries with limited healthcare resources, highlighting global health disparities [64, 65]. The journey of CaP diagnosis and treatment brings forth various physical and emotional changes, necessitating adjustment [66, 67]. Response shift theory explains these psychological adjustments and emphasizes the importance of returning to pre-diagnosis activities to foster a sense of normalcy [68, 69]. Response shift theory is a relevant framework for understanding how individuals cope, reconsider, and reframe their experiences following significant life events, such as a cancer diagnosis [68, 69]. Integrating this theory into clinical practice can, therefore, aid healthcare professionals in providing better support to CaP patients, promoting their adjustment and emotional well-being.

Engaging in advocacy activities instilled a sense of purpose and motivated some men to prioritize their health. Further research is warranted to explore the experiences of men who become advocates and understand the factors contributing to their engagement in advocacy activities. Investigating the pathways and motivations for advocacy can provide valuable insights for developing targeted interventions and support programs to empower men to become advocates for their health and the broader CaP community.

Cancer survivors display a natural progression towards advocacy, a phenomenon observed in other studies [46, 70], where personal experiences with cancers transmute into a deep commitment to community awareness. This transformative process underscores the therapeutic value of helping others as part of the individual’s healing process. Further research is warranted to explore the experiences of men who become advocates and to better understand the factors that lead to engagement in advocacy activities. Investigating the pathways and motivations for advocacy can provide valuable insights for developing targeted interventions and support programs to empower men to become advocates for their health and the broader CaP community.

Managing the fear of recurrence was identified as a vital component of the post-treatment phase, aligning with the complex survivorship model proposed by Koch et al. [71]. While some articulate their anxieties, seeking solidarity and reassurance, others silently navigate this journey. This internally held fear is consistent with the findings from Skaali et al. [72], who reported its prevalence among cancer survivors. The proactiveness of health monitoring post-diagnosis and treatment, like regular PSA screening, mirrors the recommended practices for long-term surveillance in cancer survivorship guidelines [73]. The fear of recurrence also guided treatment decisions among participants. In this study, more than half of the men were diagnosed at early stages (Stage I or II), with radiotherapy emerging as the most common form of treatment [74, 75]. Opting for radiotherapy primarily, these individuals prioritized treatments expected to offer the highest chance of survival in the event of a recurrence. This strategic approach to treatment reflects the enduring impact the fear of recurrence has on the decisions and long-term emotional state of survivors. Healthcare providers must understand these fears to guide treatment discussions and offer comprehensive care that includes psychological support. Addressing such emotional factors is crucial for informed decision-making and could improve adherence to post-treatment care and the overall well-being of survivors.

The use of grounded theory in our study presents both imitations and strengths. A limitation arises from the subjective nature of data collection, analysis, and interpretation inherent in qualitative research, where the researcher’s bias can influence the analysis. Also, we did not include perspectives from spouses and other caregivers, whose experiences may provide additional robustness to the results reported in the study. Given the use of theoretical sampling in this study, a method intrinsic to grounded theory, it is possible that our findings may not be representative of the entire Black CaP survivor population. Theoretical sampling focuses on data richness rather than demographic representativeness, which can limit the generalizability of the results. Participants were also selected based on their potential to inform theory development, so they may not reflect the broader experiences of all BM with CaP, particularly those from different socioeconomic backgrounds, regions, or medical histories. Also, while significant efforts were made, specific culture-specific interpretations and nuances may have been lost in translation. Despite these limitations, the use of grounded theory allowed for constructing a rich, bottom-up understanding of the complex phenomena of interest – experiences of Black CaP survivors. By focusing on the processes and experiences of these men, we unveiled the in-depth perspectives, behaviors, and social processes that might remain obscured under more traditional, quantitative research approaches. This methodology is particularly adept at uncovering the intricate, often personal pathways that Black CaP survivors navigate, providing crucial qualitative insights that can inform more culturally sensitive survivorship care models and targeted interventions.

## CONCLUSION

Our study findings highlight the complex journey of ethnically diverse Black CaP survivors from pre-diagnosis awareness through to adapting post-treatment life, introducing the “**Journeying through Unfamiliar Terrain**” model, encompassing three phases and 11 sub-phases. The Navigating Phase is a unique contribution to research, which sheds light on factors shaping awareness, healthcare behaviors, and cultural perceptions regarding CaP within Black communities. The findings emphasize the critical role of awareness campaigns, timely diagnoses, and culturally sensitive interventions.

The study underscores the importance of post-diagnosis coping tools like spirituality and support networks, and the impact of treatment side effects on survivors’ lives. It also highlights the enduring fear of recurrence and survivors’ progression towards advocacy. Addressing these concerns, along with the psychological impact of post-treatment life, is critical for comprehensive survivorship care. Our study is a foundation for culturally sensitive survivorship care, emphasizing patient-centered healthcare approaches. It is also a valuable resource for developing future interventions and expanding the dialogue in this critical area of survivorship research, especially for newly diagnosed ethnically diverse CaP survivors.

## Figures and Tables

**Table 1:** The semi-structured interview guide

Sample topics from an initial set of interviews
Background and Identity
Personal and cultural background
Perceptions about prostate cancer within their cultural group
Demographic details
Pertinent medical history and presence of other comorbidities
Immigration experiences (for foreign-born BM)
Diagnosis and Treatment
Reactions and emotions upon diagnosis
Impact of relationships with healthcare providers
Process of deciding on treatment
Discussions about treatment options and providers involved.
Satisfaction or regrets with the chosen treatment
Experiences and expectations during treatment
Survivorship Challenges
Impact of prostate cancer on life post-treatment
Coping strategies and available support/services
Spousal involvement
Uptake of psychosocial support
Revised topics based on emerging categories
Cultural influences on this reluctance and addressing associated stigma.
Side-effects from therapy, including impact on functionality based on types of treatment undergone.
Lifestyle adjustments post-diagnosis (diet, exercise, habits)
Role of church and involvement in social groups for support
The importance of relationships with other cancer survivors and their impact on healthcare decisions.
Experiences with their primary care providers, including their ethnic composition, and how these interactions affect CaP outcomes.
Analyzing differences in grooming processes, trust in the healthcare system, and societal norms that may deter men from seeking medical care.
Moving from CaP survivor to advocate.
Defining survivorship

**Table 2:** Clinical and Sociodemographic Characteristics of Participants (n = 34)

Characteristics	Number (%)
Age, years *(range, mean ± SD)*	49–84 (66 ±8)
Age at diagnosis, years *(range, mean ± SD)*	42–79 (58 ±8)
Stage of diagnosis *(%)*	
Stage I	12 (35.3)
Stage II	6 (17.6)
Stage III	3 (8.8)
Stage IV	2 (5.9)
Unsure	11 (32.4)
Ethnicity *(%)*	
Native-Born Black Men (NBBM)	17 (50.0)
African-Born Black Men (ABBM)	11 (32.4)
Caribbean-Born Black Men (CBBM)	6 (17.6)
Length of stay in the US if ABBM or CBBM (years) *(range, mean ± SD)*	2–55 (32 ±15)
Current treatment *(%)*	
Yes	24 (70.6)
No	10 (29.4)
Treatment type *(%)*	
Radiotherapy	19 (55.9)
Radical prostatectomy	11 (32.4)
Hormonal	6 (17.6)
Active surveillance	5 (14.7)
Chemotherapy	2 (5.9)
Marital status *(%)*	
Married	26 (76.5)
Single/Divorced/Widowed/Separated	8 (23.5)
Income *(%)*	
< $50k	12 (35.3)
$50,001-$75,000	10 (29.4)
>$75k	12 (35.3)
Family History *(%)*	
Yes	14 (41.2)
No	14 (41.2)
Unknown/Unsure	6 (17.6)
General Health *(%)*	
Fair	6 (17.6)
Good	24 (70.6)
Excellent	4 (11.8)
Social Support *(%)*	
None of the time	1 (2.9)
A little of the time	1 (2.9)
Some of the time	3 (8.8)
Most of the time	9 (26.5)
All of the time	20 (58.8)
Health insurance *(%)*	
Private insurance	22 (64.7)
Medicare	7 (20.6)
Medicaid	4 (11.8)
Other	8 (23.5)
No insurance/Self-pay	1 (2.9)
Education *(%)*	
High school graduate or less/GED	4 (11.7)
Some college or technical school	12 (35.3)
College graduate	10 (29.4)
Postgraduate	8 (23.5)
Religious Affiliation *(%)*	
Christian (all denominations)	30 (88.2)
No religion/atheist/agnostic	3 (8.8)
Muslim	1 (2.9)

## Data Availability

Relevant data generated and analyzed during this study are included in this published article.
